# Synthesis and biological evaluation of novel 7-hydroxy-4-phenylchromen-2-one–linked to triazole moieties as potent cytotoxic agents

**DOI:** 10.1080/14756366.2017.1344982

**Published:** 2017-08-09

**Authors:** Chuan-Feng Liu, Qing-Kun Shen, Jia-Jun Li, Yu-Shun Tian, Zheshan Quan

**Affiliations:** Key Laboratory of Natural Resources and Functional Molecules of the Changbai Mountain, Affiliated Ministry of Education, College of Pharmacy, Yanbian University, Yanji, PR China

**Keywords:** Coumarins, triazoles, anticancer, synthesis

## Abstract

A new series of novel 7-hydroxy-4-phenylchromen-2-one (**1a**)–linked 1,2,4-triazoles were synthesised using a click chemistry approach. All derivatives were subjected to 3-(4,5-dimethylthiazol-yl)-diphenyl tetrazolium bromide (MTT) cytotoxicity screening against a panel of six different human cancer cell lines (AGS, MGC-803, HCT-116, A-549, HepG2, and HeLa) to assess their cytotoxic potential. Among the tested molecules, some of the analogues showed better cytotoxic activity than that shown by the 7-hydroxy-4-phenylchromen-2-one (**1a**). Of the synthesised 1,2,4-triazoles,the 7-((4-(4-Chlorophenyl)-4H-1,2,4-triazol-3-yl)methoxy)-4-phenyl-2H-chromen-2-one (**4d)** showed the best activity, with an IC_50_ of 2.63 ± 0.17 µM against AGS cells. Further flow cytometry assays demonstrated that compound **4d** exerts its antiproliferative effects by arresting cells in the G2/M phase of the cell cycle and by inducing apoptosis. Collectively, our results indicate that the 1,2,4-triazole derivatives have a significantly stronger antitumour activity than 1,2,3-triazole derivatives. Most of the compounds exhibited better antitumour activity than the positive control drug 5-fluorouracil.

## Introduction

Coumarins (2H-1-benzopyran-2-ones) occupy an important place in the realm of natural products. They have diverse pharmacological properties, such as anti-inflammatory, antiviral, antimicrobial, anticancer^1^, antithrombotic, antioxidant, anticholinesterase, and antituberculosis effects. Among the natural coumarins, aesculetin (6,7-dihydroxycoumarin) has been reported to inhibit the proliferation of a number of human malignant cell lines *in vitro*, and has been shown to have activity against several types of animal tumours^2^. Coumarin A from *Angelica* species shows cytotoxicity against A-549 and MCF-7 cancer cells with IC_50_ values of 3.2 and 2.8 µM, respectively^3^. Xanthotoxin is a natural compound with antileukodermal activity and antitumour properties^4^. However, the extraction of these natural products from plants is time consuming and requires sophisticated instruments to obtain the pure product. As it requires simple and relatively inexpensive starting materials, the Pechmann reaction has been widely used for the syntheses of coumarins. Some new derivatives bearing a coumarin ring, including geiparvarin, auraptene, and ensaculin, are pharmacologically active agents that have been marketed and extensively used in the clinical setting^5^.

**Figure 1.**
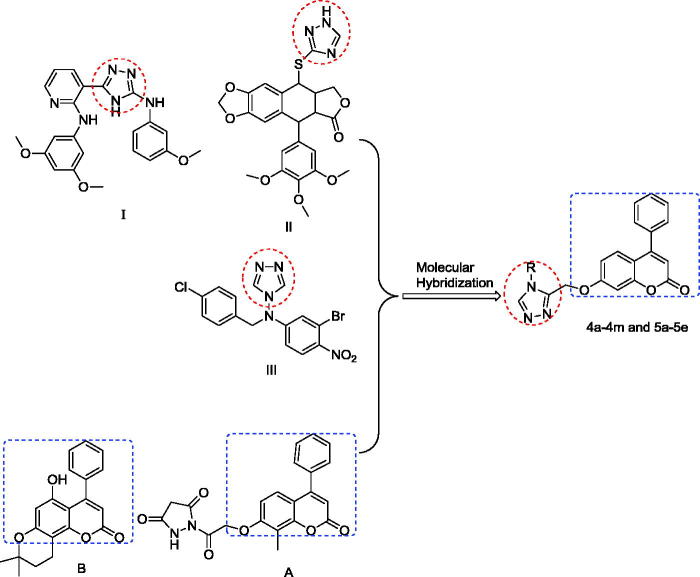
Representative examples of coumarin molecules with 1,2,4-triazoles moieties that exhibit anticancer activity.

In recent years, 7-hydroxy-4-phenylchromen-2-one and its derivatives (Figure 1) have garnered increasing interest due to their diverse pharmacological properties, and this has attracted the efforts of many medicinal chemists for further derivatisation and screening of these compounds as novel therapeutic agents. Some derivatives of 7-hydroxy-4-phenylchromen-2-one exhibited anti-inflammatory^6^, antimicrobial^7^^,^^8^, anticancer^9^^,^^10^ and antioxidant^11^ effects, and others inhibited extracellular protein^12^^,^^13^.

Substituted 1,2,4-triazoles are indispensable structural motifs of compounds (Figure 1) that display a broad spectrum of biological activities, and are widely used in organic, medicinal, and material sciences. Among them, the chemistry of 1,2,4-triazoles and their fused heterocyclic derivatives has received considerable attention owing to their synthetic and biological importance, as well as their observed antibacterial^14^, anti-HIV^15^, anticonvulsant^16^, anti-inflammatory^17^, and anticancer (compd. II)^18–20^ effects.

In this study, we focused on 7-hydroxy-4-phenylchromen-2-one (**1a**) and triazoles to design new a diverse series of triazoles derivatives based on a coumarin skeleton. Based on the above findings and inspiration from the anticancer activity of 1,2,4-triazoles, we directed this work towards the synthesis of a diverse series of novel 1,2,4-triazole (**4a–4 m**, **5a–5e**) derivatives of biological interest by using beta-keto esters as a key starting material. The potential cytotoxicity of the derivatives was evaluated *in vitro* against a panel of human tumour cell lines (Table 1).

**Table 1.** *In vitro* anticancer activity of 1a, its derivatives, and 5-fluorouracil against six cancer cell lines^a^ (IC_50_ μM^b^).
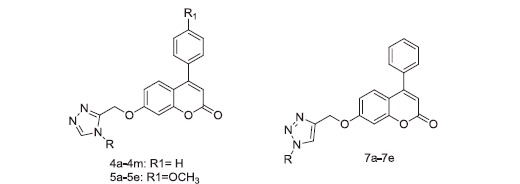

		IC_50_ (μM)					
Comp.	R	AGS	MGC	A-549	HepG2	HCT	HeLa
**4a**	–C_6_H_5_	10.60 ± 0.36	13.49 ± 0.58	26.42 ± 0.62	34.15 ± 0.24	ND	ND
**4b**	–C_6_H_4_ (*o*-Cl)	15.02 ± 0.21	18.31 ± 0.22	59.18 ± 0.66	18.75 ± 0.85	ND	ND
**4c**	–C_6_H_4_ (*m*-Cl)	17.99 ± 0.38	17.54 ± 0.43	42.56 ± 0.53	ND	**3.66 ± 0.24**	40.46 ± 0.71
**4d**	–C_6_H_4_ (*p*-Cl)	**2.63 ± 0.17**	**3.05 ± 0.29**	50.24 ± 0.51	67.83 ± 0.83	11.57 ± 0.53	13.62 ± 0.86
**4e**	–C_6_H_4_ (*p*-F)	55.96 ± 0.71	ND	ND	ND	79.16 ± 0.45	ND
**4f**	–C_6_H_4_ (*m*-Br)	22.4 ± 0.83	ND	ND	ND	12.41 ± 0.52	ND
**4g**	–C_6_H_4_ (*p*-Br)	41.36 ± 0.62	36.22 ± 0.61	33.35 ± 0.42	50.21 ± 0.36	15.58 ± 0.28	ND
**4h**	–C_6_H_4_ (*o*-CH_3_)	33.11 ± 0.36	18.93 ± 0.54	41.15 ± 0.61	8.33 ± 0.27	7.47 ± 0.69	30.93 ± 0.39
**4i**	–C_6_H_4_ (*m*-CH_3_)	61.97 ± 0.53	40.56 ± 0.29	56.77 ± 0.43	48.97 ± 0.19	7.97 ± 0.56	46.35 ± 0.43
**4j**	–C_6_H_4_ (*p*-CH_3_)	11.66 ± 0.22	8.72 ± 0.35	17.32 ± 0.63	17.71 ± 0.24	17.91 ± 0.42	7.20 ± 0.54
**4k**	–C_6_H_3_ (*2,4*-(CH_3_)_2_)	23.95 ± 0.36	32.26 ± 0.36	28.50 ± 0.87	8.45 ± 0.15	9.54 ± 0.52	20.4 ± 0.62
**4l**	–C_6_H_3_ (*2,6*-(CH_3_)_2_)	62.71 ± 0.79	43.37 ± 0.27	ND	23.41 ± 0.26	15.72 ± 0.31	57.91 ± 0.69
**4m**	–C_6_H_4_ (*p*-OCH_3_)	9.00 ± 0.64	ND	ND	ND	ND	ND
**5a**	–C_6_H_5_	16.80 ± 0.83	54.53 ± 0.42	63.72 ± 0.60	ND	98.14 ± 0.61	73.70 ± 0.76
**5b**	–C_6_H_4_ (*o*-Cl)	96.21 ± 0.26	74.50 ± 0.30	ND	ND	45.47 ± 0.49	29.39 ± 0.63
**5c**	–C_6_H_4_ (*p*-Cl)	ND	72.99 ± 0.51	ND	ND	ND	ND
**5d**	–C_6_H_4_ (*p*-F)	ND	ND	ND	ND	99.46 ± 0.28	26.23 ± 0.72
**5e**	–C_6_H_4_ (*p*-CH_3_)	ND	ND	ND	ND	ND	ND
**7a**	–C_6_H_5_	ND	ND	ND	ND	ND	ND
**7b**	–CH_2_C_6_H_5_	ND	ND	ND	ND	ND	ND
**7c**	–CH_2_C_6_H_4_*(p*-Cl)	ND	48.16 ± 0.46	ND	ND	ND	ND
**7d**	–CH_2_C_6_H_4_ (*p*-F)	ND	ND	ND	ND	94.36 ± 1.32	ND
**7e**	–CH_2_C_6_H_4_ (*p*-CH_3_)	ND	ND	ND	ND	ND	ND
**1a**		>100	>100	>100	>100	>100	>100
**5-FU**		29.61 ± 0.21	30.52 ± 0.36	23.65 ± 0.19	23.45 ± 0.37	24.80 ± 0.29	34.61 ± 0.42

Bold values signify that the bioactivity of the compound is outstanding.

a Cytotoxicity as IC_50_ for each cell line, refers to the concentration of compound which reduced by 50% the optical density of treated cells with respect to untreated cells using the MTT assay.

b Data represent the mean values of three independent determinations. ND: not determined (these compounds did not pass the preliminary screening).

## Materials and methods

### Chemistry

Melting points were determined in open capillary tubes and were uncorrected. IR spectra were recorded (in KBr) on IR Prestige-21. ^1^H-NMR and ^13^C-NMR spectra were measured on an AV-300 (Bruker, Switzerland), and all chemical shifts were given in ppm relative to TMS. High-resolution mass spectra were measured using a matrix-assisted laser desorption/ionisation (MALDI)-time of flight (TOF)/TOF mass spectrometer (Bruker Daltonik, Germany). The major chemicals were purchased from Aldrich Chemical Corporation (Milwaukee, WI). All other chemicals were of analytical grade.

### General procedure for the synthesis of compound 1a,b

We added 30 mmol of properly substituted resorcinol to 30 ml of perchloric acid. Then 30 mmol of β-keto ester was added and the reaction mixture was stirred at room temperature until TLC showed that the resorcinol had reacted. The mixture was poured into 250 ml of cold water and the precipitate was filtered and dried at room temperature and recrystallised from ethanol^21^.

#### 7-Hydroxy-4-phenyl-2H-chromen-2-one (1a)

mp 253–255 °C; yield 96%. ^1^H-NMR (DMSO-d_6_, 300 MHz): *δ* 6.27 (s, 1H, C=CH), 6.74 (s, 2H, C=CH), 7.23 (s, 1H, C=CH), 7.49–7.58 (m, 5H, Ar-H), 10.48 (s, 1H, –OH).

#### 7-Hydroxy-4-(4-methoxyphenyl)-2H-chromen-2-one (1b)

mp 263–265 °C; yield 94%. ^1^H-NMR (DMSO-d_6_, 300 MHz): *δ* 3.84 (s, 3H, –OCH_3_) 6.11 (s, 1H, C=CH), 6.77 (d, 1H, *J* = 2.4 Hz, C=CH), 6.80 (s, 1H, C=CH), 7.09 (s, 1H, C=CH), 7.12–7.49 (m, 4H, Ar-H), 10.68 (s, 1H, –OH).

### General procedure for the synthesis of compounds 2a,b

To a solution of compound **1a,b** (10 mmol) in acetone (30 ml) was added ethyl bromoacetate (11 mmol) and K_2_CO_3_ (2.76 g, 12 mmol). After the reaction mixture was stirred at 56 °C for 3 h, the mixture was added into 30 ml of ice-water and filtered to obtain a white solid. Finally, the compounds were purified by chromatography on silica via elution with a gradient of methanol/dichloromethane (1:80) to give compounds **2a,b**^22^ as solids.

#### Ethyl 2-((2-oxo-4-phenyl-2H-chromen-7-yl)oxy)acetate (2a)

mp 140–142 °C; yield 78%. ^1^H-NMR (DMSO-d_6_, 300 MHz): *δ* 1.20 (*t*, 3H, *J* = 7.2 Hz, –CH_3_) 4.15 (q, 2H, *J* = 7.2 Hz, –CH_2_–) 4.95 (s, 2H, –OCH_2_–) 6.27 (s, 1H, C=CH), 6.95 (dd, 1H, *J* = 8.7, 2.4 Hz, C=CH), 7.09 (d, 1H, *J* = 2.4 Hz, C=CH), 7.11 (s, 1H, *J* = 8.7 Hz, C=CH), 7.52–7.59 (m, 5H, Ar-H).

#### Ethyl 2-((4-(4-methoxyphenyl)-2-oxo-2H-chromen-7-yl)oxy)acetate (2b)

mp 128–130 °C; yield 73%. ^1^H-NMR (DMSO-d_6_, 300 MHz): δ 1.20 (t, 3H, *J* = 6.9 Hz, –CH_3_) 3.84 (s, 3H, –OCH_3_) 4.15 (q, 2H, *J* = 7.2 Hz, –CH_2_–) 4.95 (s, 2H, –OCH_2_–6.22 (s, 1H, C=CH), 6.96 (dd, 1H, *J* = 9.0, 2.4 Hz, C=CH), 7.09 (d, 1H, *J* = 2.4 Hz, C=CH), 7.11 (s, 1H, C=CH), 7.14–7.51 (m, 4H, Ar-H).

### General procedure for the synthesis of compounds 3a,b

To a solution of compounds **2a,b** (10 mmol) in ethyl alcohol (30 ml) was added hydrazine hydrate (11 mmol). After the reaction mixture was stirred at 78 °C for 4 h, the mixture was added to 30 ml of ice-water and filtered to obtain a pink solid^23^.

#### 2-((2-Oxo-4-phenyl-2H-chromen-7-yl)oxy)acetohydrazide (3a)

mp 188–190 °C; yield 80%. ^1^H-NMR (DMSO-d_6_, 300 MHz): *δ* 4.37 (s, 2H, –NH_2_), 4.64 (s, 2H, –OCH_2_–), 6.26 (s, 1H, C=CH), 6.97 (d, 1H, *J* = 9.0 Hz, C=CH), 7.08 (s, 1H, C=CH), 7.35 (d, 1H, *J* = 8.7 Hz, C=CH), 7.54–7.56 (m, 5H, Ar-H), 9.45 (s, 1H, –NH–).

#### 2-((4-(4-Methoxyphenyl)-2-oxo-2H-chromen-7-yl)oxy)acetohydrazide (3b)

mp 148–150 °C; yield 83%. ^1^H-NMR (DMSO-d_6_, 300 MHz): *δ* 3.84 (s, 3H, –OCH_3_) 4.37 (s, 2H, –NH_2_), 4.64 (s, 2H, –OCH_2_–), 6.22 (s, 1H, C=CH), 6.97 (dd, 1H, *J* = 9.0, 2.4 Hz, C=CH), 7.06 (d, 1H, *J* = 2.1 Hz, C=CH), 7.11 (s, 1H, C=CH), 7.14–7.51 (m, 4H, Ar-H), 9.45 (s, 1H, –NH–).

### General procedure for the synthesis of compounds 4a–4 m and 5a–5e

A mixture of **3a,b** (6 mmol) and dimethylacetal (0.72 g, 6 mmol) in acetonitrile (50 ml) was stirred at 60 °C for 1 h and then various aromatic amines (7.2 mmol) were added, followed by glacial acetic acid (6 ml). The mixture was refluxed at 120 °C for 24 h, then was cooled and concentrated under reduced pressure. The residue was extracted with ethyl acetate. The organic layer was dried over anhydrous MgSO_4_ and concentrated under reduced pressure to give the crude product as a solid. The solid could be purified by chromatography on silica by elution with a gradient of methanol/dichloromethane (1:80) to obtain compounds **4a–4 m** and **5a–5e**.

#### 4-Phenyl-7-((4-phenyl-4*H*-1,2,4-triazol-3-yl)methoxy)-*2H*-chromen-2-one (4a)

mp 138–140 °C; yield 54%. ^1^H-NMR (DMSO-d_6_, 300 MHz): *δ* 5.39 (s, 2H, –OCH_2_–), 6.27 (s, 1H, C=CH), 6.87 (dd, 1H, *J* = 9.0, 2.4 Hz, C=CH), 7.20 (d, 1H, *J* = 2.4 Hz, C=CH), 7.37 (d, 1H, *J* = 8.7 Hz, C=CH), 7.49–7.58 (m, 10H, Ar-H), 8.92 (s, 1H, Triazole-H)^13^. C-NMR (CD-Cl_3_, 75 MHz): *δ* 59.62, 102.62, 111.92, 112.33, 113.35, 125.16, 128.16, 128.71, 128.55, 128.85, 128.92, 133.20, 135.11, 144.52, 149.11, 155.39, 155.48, 160.18, and 160.67. IR (KBr) cm^−1^: 1726. **ESI-HRMS** (*m/z*): calcd for C_24_H_17_N_3_O_3_^+^ [M + H]^+^: 396.1343; found: 396.1339.

#### 7-((4-(2-Chlorophenyl)-4*H*-1,2,4-triazol-3-yl)methoxy)-4-phenyl-2*H*-chromen-2-one (4b)

mp 106–108 °C; yield 62%. ^1^H-NMR (CD-Cl_3_, 300 MHz): *δ* 5.30 (s, 2H, –OCH_2_–), 6.21 (s, 1H, C=CH), 6.75 (d, 1H, *J* = 2.7 Hz, C=CH), 6.79 (s, 1H, C=CH), 7.34 (s, 1H, C=CH), 7.37–7.62 (m, 9H, Ar-H), 8.28 (s, 1H, Triazole-H). ^13^C-NMR (CD-Cl_3_, 75 MHz): *δ* 60.28, 102.68, 111.78, 112.41, 113.42, 128.18, 128.26, 128.30, 128.86, 129.71, 130.74, 131.09, 131.41, 131.80, 135.21, 155.53, 160.25, and 160.80. IR (KBr) cm^−1^: 1702. **ESI-HRMS** (*m/z*): calcd for C_24_H_17_ClN_3_O_3_^+^ [M + H]^+^: 430.0953; found: 430.0946.

#### 7-((4-(3-Chlorophenyl)-4*H*-1,2,4-triazol-3-yl)methoxy)-4-phenyl-2*H*-chromen-2-one (4c)

mp 138–140 °C; yield 64%. ^1^H-NMR (CD-Cl_3_, 300 MHz): *δ* 5.28 (s, 2H, –OCH_2_–), 6.24 (s, 1H, C=CH), 6.87 (dd, 1H, *J* =9.0, 2.4 Hz, C=CH), 6.94 (d, 1H, *J* = 2.4 Hz, C=CH), 7.33 (d, 1H, *J* =12.3 Hz, C=CH), 7.40–7.53 (m, 9H, Ar-H), 8.36 (s, 1H, Triazole-H). ^13^C-NMR (CD-Cl_3_, 75 MHz): *δ* 59.67, 102.58, 111.96, 112.39, 113.43, 123.51, 125.69, 128.18, 128.73, 129.58, 130.08, 130.96, 134.20, 135.08, 135.50, 144.34, 149.00, 155.37, 155.50, 160.01, and 160.62. IR (KBr) cm^−1^: 1720. **ESI-HRMS** (*m/z*): calcd for C_24_H_17_ClN_3_O_3_^+^ [M + H]^+^: 430.0953; found: 430.0946.

#### 7-((4-(4-Chlorophenyl)-4*H*-1,2,4-triazol-3-yl)methoxy)-4-phenyl-2*H*-chromen-2-one (4d)

mp 161–163 °C; yield 59%. ^1^H-NMR (CD-Cl_3_, 300 MHz): *δ* 5.26 (s, 2H, –OCH_2_–), 6.24 (s, 1H, C=CH), 6.87 (d, 1H, *J* = 8.7 Hz, C=CH), 6.95 (s, 1H, C=CH), 7.38 (s, 1H, C=CH), 7.41–7.54 (m, 9H, Ar-H), 8.36 (S, 1H, Triazole-H). ^13^C-NMR (CD-Cl_3_, 75 MHz): *δ* 59.76, 102.61, 112.14, 112.38, 113.45, 126.81, 128.32, 128.86, 129.72, 129.71, 130.22, 131.85, 135.17, 135.98, 144.70, 149.30, 155.59, 160.24, and 160.75. IR (KBr) cm^−1^: 1714. ESI-HRMS (*m/z*): calcd for C_24_H_17_ClN_3_O_3_^+^ [M + H]^+^: 430.0953; found: 430.0946.

#### 7-((4-(4-Fluorophenyl)-4*H*-1,2,4-triazol-3-yl)methoxy)-4-phenyl-2*H*-chromen-2-one (4e)

mp 190–192 °C; yield 61%. ^1^H-NMR (DMSO-d_6_, 300 MHz): *δ* 5.32 (s, 2H, –OCH_2_–), 6.17 (s, 1H, C=CH), 6.85 (dd, 1H, *J* = 8.7, 2.1 Hz, C=CH), 7.08 (s, 1H, C=CH), 7.24 (s, 1H, C=CH), 7.29–7.53 (m, 9H, Ar-H), 8.60 (s, 1H, Triazole-H). ^13^C-NMR (CD-Cl_3_-DMSO-d_6_, 75 MHz): δ 64.88, 107.35, 116.97, 117.49, 117.88, 121.49, 121.80, 132.58, 132.70, 132.99, 133.25, 133.79, 134.60, 140.04, 150.14, 154.08, 160.22, 160.42, 165.00, 165.37 (d, *J*_c–f_ = 33.75 Hz). IR (KBr) cm^−1^: 1741. ESI-HRMS (*m/z*): calcd for C_24_H_17_FN_3_O_3_^+^ [M + H]^+^: 414.1248; found: 414.1235.

#### 7-((4-(3-Bromophenyl)-4H-1,2,4-triazol-3-yl)methoxy)-4-phenyl-2H-chromen-2-one (4f)

mp 168–170 °C; yield 58%. ^1^H-NMR (DMSO-d_6_, 300 MHz): *δ* 5.25 (s, 2H, –OCH_2_–), 6.21 (s, 1H, C=CH), 6.85 (dd, 1H, *J* = 9.0, 2.4 Hz, C=CH), 6.93 (d, 1H, *J* = 2.4 Hz, C=CH), 7.38 (s, 1H, C=CH), 7.41–7.66 (m, 9H, Ar-H), 8.35 (s, 1H, Triazole-H). ^13^C-NMR (CD-Cl_3_-DMSO-d_6_, 75 MHz): *δ* 64.76, 107.35, 117.02, 117.18, 118.12, 127.79, 128.99, 133.09, 133.36, 133.67, 134.51, 136.12, 137.64, 139.35, 139.94, 160.38, 165.09, and 165.30. IR (KBr) cm^−1^: 1729. ESI-HRMS (*m/z*): calcd for C_24_H_17_BrN_3_O_3_^+^ [M + H]^+^: 474.0448; found: 474.0437.

#### 7-((4-(4-Bromophenyl)-4*H*-1,2,4-triazol-3-yl)methoxy)-4-phenyl-2*H*-chromen-2-on (4g)

mp 96–98 °C; yield 64%. ^1^H-NMR (CD-Cl_3_, 300 MHz): *δ* 5.25 (s, 2H, –OCH_2_–), 6.26 (s, 1H, C=CH), 6.88 (dd, 1H, *J* =9.0, 2.4 Hz, C=CH), 6.95 (d, 1H, *J* = 2.4 Hz, C=CH), 7.30 (s, 1H, C=CH), 7.33–7.70 (m, 9H, Ar-H), 8.34 (s, 1H, Triazole-H). ^13^C-NMR (CD-Cl_3_, 75 MHz): *δ* 59.56, 102.54, 111.89, 112.43, 113.46, 124.03, 126.84, 128.20, 128.26, 128.75, 129.59, 132.18, 133.15, 135.09, 144.37, 149.04, 155.40, 155.51, 160.03, and 160.65. IR (KBr) cm^−1^: 1718. **ESI-HRMS** (*m/z*): calcd for C_24_H_17_BrN_3_O_3_^+^ [M + H]^+^: 474.0448; found: 474.0437.

#### 4-Phenyl-7-((4-(o-tolyl)-4*H*-1,2,4-triazol-3-yl)methoxy)-2*H*-chromen-2-on (4h)

mp 112–114 °C; yield 63%. ^1^H-NMR (CD-Cl_3_, 300 MHz): *δ* 2.22 (s, 3H, –CH_3_), 5.31 (s, 2H, –OCH_2_–), 6.35 (s, 1H, C=CH), 6.91 (d, 1H, *J* = 2.7 Hz, C=CH), 6.95 (d, 1H, *J* = 1.8 Hz, C=CH), 7.36 (d, 1H, *J* = 7.8 Hz, C=CH), 7.41–7.66 (m, 9H, Ar-H), 8.38 (s, 1H, Triazole-H). ^13^C-NMR (CD-Cl_3_, 75 MHz): *δ* 17.17, 59.74, 102.48, 111.68, 112.29, 113.25, 127.10, 127.12, 128.10, 128.16, 128.71, 129.55, 130.46, 131.35, 132.10, 135.02, 135.11, 144.76, 149.55, 155.36, 155.43, 160.24, and 160.64. IR (KBr) cm^−1^: 1720. ESI-HRMS (*m/z*): calcd for C_25_H_20_N_3_O_3_^+^ [M + H]^+^: 410.1499; found: 410.1489.

#### 4-Phenyl-7-((4-(m-tolyl)-4*H*-1,2,4-triazol-3-yl)methoxy)-2*H*-chromen-2-one (4i)

mp 150–152 °C; yield 66%. ^1^H-NMR (CD-Cl_3_, 300 MHz): *δ* 2.41 (s, 3H, –CH_3_), 5.24 (s, 2H, –OCH_2_–), 6.24 (s, 1H, C=CH), 6.88 (d, 1H, *J* = 2.4 Hz, C=CH), 6.95 (s, 1H, C=CH), 7.21 (d, 1H, *J* = 5.1 Hz, C=CH), 7.32–7.53 (m, 9H, Ar-H), 8.33 (s, 1H, Triazole-H). ^13^C-NMR (CD-Cl_3_, 75 MHz): *δ* 21.17, 59.66, 102.63, 111.95, 112.35, 113.34, 122.18, 125.80, 128.18, 128.73, 129.56, 129.66, 130.57, 133.09, 135.14, 140.23, 144.51, 149.10, 155.41, 155.51, 160.24, and 160.68. IR (KBr) cm^−1^: 1720. ESI-HRMS (*m/z*): calcd for C_25_H_20_N_3_O_3_^+^ [M + H]^+^: 410.1499; found: 410.1489.

#### 4-Phenyl-7-((4-(p-tolyl)-4*H*-1,2,4-triazol-3-yl)methoxy)-2*H*-chromen-2-one (4j)

mp 84–86 °C; yield 68%. ^1^H-NMR (DMSO-d_6_, 300 MHz): *δ* 2.34 (s, 3H, –CH_3_), 5.36 (s, 2H, –OCH_2_–6.27 (s, 1H, C=CH), 6.92 (dd, 1H, *J* = 8.7, 2.4 Hz, C=CH), 7.19 (d, 1H, *J* = 2.4 Hz, C=CH), 7.31 (d, 1H, *J* = 6.9 Hz, C=CH), 7.33–7.58 (m, 9H, Ar-H), 8.87 (s, 1H, Triazole-H). ^13^C-NMR (DMSO-d_6_, 75 MHz): *δ* 21.03, 49.06, 60.37, 102.81, 112.31, 112.96, 113.32, 125.52, 128.89, 129.33, 130.16, 130.61, 131.47, 135.31, 139.54, 145.85, 149.29, 155.43, 155.62, 160.31, and 160.83. IR (KBr) cm^−1^: 1705. ESI-HRMS (*m/z*): calcd for C_25_H_20_N_3_O_3_^+^ [M + H]^+^: 410.1499; found: 410.1489.

#### 7-((4-(2,4-Dimethylphenyl)-4*H*-1,2,4-triazol-3-yl)methoxy)-4-phenyl-2*H*-chromen-2-one (4k)

mp 152–154 °C; yield 62%. ^1^H-NMR (CD-Cl_3_, 300 MHz): *δ* 2.03 (s, 3H, –CH_3_), 2.38 (s, 3H, –CH_3_), 5.15 (s, 2H, –OCH_2_–), 6.20 (s, 1H, C=CH), 6.79 (d, 1H, *J* = 2.1 Hz, C=CH), 6.82 (s, 1H, C=CH), 7.11 (s, 1H, C=CH), 7.17–7.51 (m, 8H, Ar-H), 8.21 (s, 1H, Triazole-H). ^13^C-NMR (CD-Cl_3_, 75 MHz): *δ* 17.10, 21.06, 59.80, 102.69, 111.79, 112.44, 113.40, 126.94, 127.80, 128.14, 128.23, 128.77, 129.58, 132.03, 134.71, 135.30, 140.78, 144.96, 149.76, 155.42, 155.59, 160.43, and 160.66. IR (KBr) cm^−1^: 1723. ESI-HRMS (*m/z*): calcd for C_26_H_22_N_3_O_3_^+^ [M + H]^+^: 424.1656; found: 424.1645.

#### 7-((4-(2,6-Dimethylphenyl)-4*H*-1,2,4-triazol-3-yl)methoxy)-4-phenyl-2*H*-chromen-2-one (4l)

mp 160–162 °C; yield 58%. ^1^H-NMR (CD-Cl_3_, 300 MHz): *δ* 2.01 (s, 6H, –CH_3_), 5.10 (s, 2H, –OCH_2_–), 6.22 (s, 1H, C=CH), 6.77 (d, 1H, *J* =2.7 Hz, C=CH), 6.81 (d, 1H, *J* = 1.8 Hz, C=CH), 7.19 (s, 1H, C=CH), 7.30–7.54 (m, 8H, Ar-H), 8.18 (s, 1H, Triazole-H). ^13^C-NMR (CD-Cl_3_, 75 MHz): *δ* 17.52, 60.10, 102.68, 111.62, 112.69, 113.55, 128.26, 128.31, 128.84, 128.88, 129.65, 130.32, 135.70, 144.44, 155.46, 155.66, 160.52, and 160.72. IR (KBr) cm^−1^: 1723. ESI-HRMS (*m/z*): calcd for C_26_H_22_N_3_O_3_^+^ [M + H]^+^: 424.1656; found: 424.1645.

#### 7-((4-(4-Methoxyphenyl)-4*H*-1,2,4-triazol-3-yl)methoxy)-4-phenyl-2*H*-chromen-2-one (4m)

mp 158–160 °C; yield 67%. ^1^H-NMR (CD-Cl_3_, 300 MHz): *δ* 3.87 (s, 3H, –OCH_3_), 5.23 (s, 2H, –OCH_2_–), 6.25 (s, 1H, C=CH), 6.90 (d, 1H, *J* = 2.4 Hz, C=CH), 6.93 (s, 1H, C=CH), 7.00 (s, 1H, C=CH), 7.03–7.54 (m, 9H, Ar-H), 8.31 (s, 1H, Triazole-H). ^13^C-NMR (CD-Cl_3_, 75 MHz): δ 55.69, 59.70, 102.76, 112.09, 112.46, 113.47, 115.06, 125.83, 126.81, 128.33, 128.87, 129.71, 135.29, 144.97, 149.57, 155.59, 155.65, 160.41, 160.60, and 160.87. IR (KBr) cm^−1^: 1723. ESI-HRMS (*m/z*): calcd for C_25_H_19_N_3_O_4_^+^ [M + H]^+^: 426.1448; found: 426.1439.

#### 4-(4-Methoxyphenyl)-7-((4-phenyl-4*H*-1,2,4-triazol-3-yl)methoxy)-2*H*-chromen-2-one (5a)

mp 184–186 °C; yield 56%. ^1^H-NMR (CD-Cl_3_, 300 MHz): *δ* 3.90 (s, 3H, –OCH_3_), 5.27 (s, 2H, –OCH_2_–), 6.21 (s, 1H, C=CH), 6.89 (dd, 1H, *J* = 6 Hz, *J* = 2 Hz, C=CH), 6.91 (d, 1H, *J* = 3 Hz, C=CH), 7.03 (s, 1H, C=CH), 7.06–7.56 (m, 8H, Ar-H), 8.36 (s, 1H, Triazole-H). ^13^C-NMR (CD-Cl_3_, 75 MHz): *δ* 55.37, 59.85, 102.86, 111.86, 112.00, 113.70, 114.30, 125.29, 127.54, 128.24, 129.75, 129.91, 129.98, 133.14, 144.56, 149.25, 155.09, 155.67, 160.22, and 160.82. IR (KBr) cm^−1^: 1702. ESI-HRMS (*m/z*): calcd for C_25_H_19_N_3_O_4_^+^ [M + H]^+^: 426.1448; found: 426.1439.

#### 7-((4-(2-Chlorophenyl)-4*H*-1,2,4-triazol-3-yl)methoxy)-4-(4-methoxyphenyl)-2*H*-chromen-2-one (5b)

mp 172–174 °C; yield 59%. ^1^H-NMR (CD-Cl_3_, 300 MHz): *δ* 3.91 (s, 3H, –OCH_3_), 5.28 (s, 2H, –OCH_2_–), 6.21 (s, 1H, C=CH), 6.78 (s, 1H, C=CH), 6.81 (s, 1H, C=CH), 7.03 (s, 1H, C=CH), 7.06–7.64 (m, 8H, Ar-H), 8.29 (s, 1H, Triazole-H). ^13^C-NMR (CD-Cl_3_, 75 MHz): *δ* 55.27, 60.21, 102.64, 111.46, 111.79, 113.49, 114.19, 127.36, 127.96, 128.09, 128.68, 129.63, 130.58, 131.06, 131.32, 131.55, 144.63, 149.52, 154.97, 155.45, 160.04, and 160.66. IR (KBr) cm^−1^: 1723. ESI-HRMS (*m/z*): calcd for C_25_H_19_ClN_3_O_4_^+^ [M + H]^+^: 460.1059; found: 460.1046.

#### 7-((4-(4-Chlorophenyl)-4*H*-1,2,4-triazol-3-yl)methoxy)-4-(4-methoxyphenyl)-2*H*-chromen-2-one (5c)

mp 189–191 °C; yield 53%. ^1^H-NMR (CD-Cl_3_, 300 MHz): *δ* 3.91 (s, 3H, –OCH_3_), 5.26 (s, 2H, –OCH_2_–), 6.23 (s, 1H, C=CH), 6.89 (d, 1H, *J* = 9.0 Hz, C=CH), 6.92 (s, 1H, C=CH), 7.04 (s, 1H, C=CH), 7.07–7.55 (m, 8H, Ar-H), 8.35 (s, 1H, Triazole-H). ^13^C-NMR (CD-Cl_3_, 75 MHz): *δ* 55.31, 59.57, 102.54, 111.76, 111.84, 113.57, 114.18, 126.60, 127.31, 128.25, 129.70, 130.13, 131.68, 136.00, 144.42, 149.11, 155.04, 155.53, 159.94, 160.68, and 160.78. IR (KBr) cm^−1^: 1703. ESI-HRMS (*m/z*): calcd for C_25_H_19_ClN_3_O_4_^+^ [M + H]^+^: 460.1059; found: 460.1046.

#### 7-((4-(4-Fluorophenyl)-4*H*-1,2,4-triazol-3-yl)methoxy)-4-(4-methoxyphenyl)-2*H*-chromen-2-one (5d)

mp 122–124 °C; yield 50%. ^1^H-NMR (CD-Cl_3_, 300 MHz): *δ* 3.91 (s, 3H, –OCH_3_), 5.26 (s, 2H, –OCH_2_–), 6.23 (s, 1H, C=CH), 6.88 (dd, 1H, *J* = 9.0, 2.1 Hz, C=CH), 6.93 (s, 1H, C=CH), 7.04 (s, 1H, C=CH), 7.06–7.50 (m, 8H, Ar-H), 8.35 (s, 1H, Triazole-H). ^13^C-NMR (CD-Cl_3_, 75 MHz): *δ* 55.37, 59.76, 102.72, 111.81, 112.00, 113.73, 114.31, 116.88, 117.18, 127.43, 127.47, 127.54, 128.30, 129.39, 129.75, 144.70, 149.42, 155.08, 155.66, 160.10, 160.76, 160.82, 161.38, and 164.71. IR (KBr) cm^−1^: 1714. ESI-HRMS (*m/z*): calcd for C_25_H_18_FN_3_O_4_^+^ [M + H]^+^: 443.1276; found: 443.1263.

#### 4-(4-Methoxyphenyl)-7-((4-(p-tolyl)-4*H*-1,2,4-triazol-3-yl)methoxy)-2*H*-chromen-2-one (5e)

mp 158–160 °C; yield 61%. ^1^H-NMR (CD-Cl_3_, 300 MHz): *δ* 2.45 (s, 3H, –CH_3_), 3.91 (s, 3H, –OCH_3_), 5.25 (s, 2H, –OCH_2_–), 6.23 (s, 1H, C=CH), 6.92 (s, 1H, C=CH), 6.94 (s, 1H, C=CH), 7.04 (s, 1H, C=CH), 7.07–7.50 (m, 8H, Ar-H), 8.33 (s, 1H, Triazole-H). ^13^C-NMR (CD-Cl_3_, 75 MHz): *δ* 21.06, 55.37, 59.79, 102.80, 111.89, 111.93, 113.59, 114.29, 125.10, 127.51, 128.21, 129.74, 130.48, 130.75, 140.21, 144.65, 149.31, 155.10, 155.64, 160.29, and 160.80. IR (KBr) cm^−1^: 1723. ESI-HRMS (*m/z*): calcd for C_26_H_21_N_3_O_4_^+^ [M + H]^+^: 439.1527; found: 439.1518.

### General procedure for the synthesis of compounds 6a

To a solution of Compound **1a** (2.38 g, 10 mmol) in acetone (30 ml) was added propargyl bromide (11 mmol) and K_2_CO_3_ (2.76 g, 12 mmol). After the reaction mixture was stirred at 56 °C for 4 h, the mixture was added into 50 ml of ice-water and filtered to obtain a light white solid. Finally, they can be purified by chromatography on silica eluting with a gradient of methanol/dichloromethane (1:80) to give the compound **6a**^24^ as solid. mp 108–110 °C; yield 71%. ^1^H-NMR (DMSO-d_6_, 300 MHz): *δ* 3.65 (s, 1H, CH) 4.97 (s, 2H, –OCH_2_–), 6.28 (s, 1H, C=CH), 6.97 (dd, 1H, *J* = 9.0, 2.1 Hz, C=CH), 7.16 (d, 1H, *J* = 2.1 Hz, C=CH), 7.36 (s, 1H, *J* = 9.0 Hz, C=CH), 7.55–7.57 (m, 5H, Ar-H).

### General procedure for the synthesis of compounds 7a–7e

To a stirred solution of various aromatic azides (0.2 mmol) and alkyne **6a** (0.22 mmol) in t-BuOH:H_2_O (3:3 ml), CuSO_4_^.^5H_2_O (0.22 mmol), and sodium ascorbate (0.5 mmol) was added. The reaction mixture was stirred at room temperature for 3 h and then concentrated under reduced pressure. To the residue was added water (10 ml) and extracted with dichloromethane (3 × 20 ml). The combined organic phases were washed with water (20 ml), dried over anhydrous sodium sulphate and evaporated to dryness. The crude product was purified using silica gel column chromatography and eluted with methanol:dichloromethane (1:60).

#### 4-Phenyl-7-((1-phenyl-1*H*-1,2,3-triazol-4-yl)methoxy)-2*H*-chromen-2-one (7a)

mp 192–194 °C; yield 59%. ^1^H-NMR (CD-Cl_3_, 300 MHz): *δ* 5.26 (s, 2H, –OCH_2_–), 6.24 (s, 1H, C=CH), 6.87 (d, 1H, *J* = 2.4 Hz, C=CH), 6.92 (s, 1H, C=CH), 7.39 (s, 1H, C=CH), 7.42–7.56 (m, 10H, Ar-H), 8.36 (s, 1H, Triazole-H). ^13^C-NMR (CD-Cl_3_, 75 MHz): *δ* 59.65, 102.62, 111.92, 112.33, 113.35, 125.16, 128.16, 128.71, 129.55, 129.85, 129.92, 133.20, 135.11, 144.52, 149.11, 155.39, 155.48, 160.18, and 160.67. IR (KBr) cm^−1^: 1726. **ESI-HRMS** (*m/z*): calcd for C_24_H_18_N_3_O_3_^+^ [M + H]^+^: 396.1343; found: 396.1046.

#### 7-((1-Benzyl-1*H*-1,2,3-triazol-4-yl)methoxy)-4-phenyl-2*H*-chromen-2-one (7b)

mp 102–104 °C; yield 86%. ^1^H-NMR (DMSO-d_6_, 300 MHz): *δ* 5.25 (s, 2H, –OCH_2_–), 5.60 (s, 2H, –CH_2_–), 6.16 (s, 1H, C=CH), 6.89 (d, 1H, *J* = 8.7 Hz, C=CH), 7.08 (d, 1H, *J* = 6.6 Hz, C=CH), 7.33 (s, 1H, C=CH), 7.34–7.52 (m, 10H, Ar-H), 8.10 (s, 1H, Triazole-H). ^13^C-NMR (CD-Cl_3_-DMSO-d_6_, 75 MHz): *δ* 58.62, 66.90, 106.99, 116.57, 117.37, 117.59, 132.58, 132.91, 133.19, 133.31, 133.72, 133.77, 134.52, 140.10, 140.16, 160.41, 160.54, 165.28, and 166.21. IR (KBr) cm^−1^: 1723. **ESI-HRMS** (*m/z*): calcd for C_25_H_20_N_3_O_3_^+^ [M + H]^+^: 410.1499; found: 410.1491.

#### 7-((1-(4-Chlorobenzyl)-1*H*-1,2,3-triazol-4-yl)methoxy)-4-phenyl-2*H*-chromen-2-one (7c)

mp 141–143 °C; yield 81%. ^1^H-NMR (DMSO-d_6_, 300 MHz): *δ* 5.28 (s, 2H, –OCH_2_–), 5.63 (s, 2H, –CH_2_–), 6.25 (s, 1H, C=CH), 6.97 (d, 1H, *J* = 8.4 Hz, C=CH), 7.26 (s, 1H, C=CH), 7.33 (s, 1H, C=CH), 7.36–7.55 (m, 9H, Ar-H), 8.36 (s, 1H, Triazole-H). ^13^C-NMR (DMSO-d_6_, 75 MHz): *δ* 49.05, 52.01, 62.11, 102.47, 111.94, 112.43, 113.41, 125.56, 128.25, 128.87, 129.22, 129.29, 130.11, 130.39, 133.37, 135.38, 142.72, 155.52, 155.82, 160.43, and 160.55. IR (KBr) cm^−^1: 1727. ESI-HRMS (*m/z*): calcd for C_25_H_19_ClN_3_O_3_^+^ [M + H]^+^: 444.1109; found: 444.1101.

#### 7-((1-(4-Fluorobenzyl)-1*H*-1,2,3-triazol-4-yl)methoxy)-4-phenyl-2*H*-chromen-2-one (7d)

mp 138–140 °C; yield 83%. ^1^H-NMR (DMSO-d_6_, 300 MHz): *δ* 5.28 (s, 2H, –OCH_2_–), 5.62 (s, 2H, –CH_2_–), 6.26 (s, 1H, C=CH), 6.97 (dd, 1H, *J* = 9.0 Hz, *J* = 2.4 Hz, C=CH), 7.19 (s, 1H, C=CH), 7.25 (s, 1H, C=CH), 7.26–7.58 (m, 9H, Ar-H), 8.36 (s, 1H, Triazole-H). ^13^C-NMR (DMSO-d_6_, 75 MHz): *δ* 52.52, 62.12, 102.49, 111.97, 112.46, 113.46, 115.94, 116.23, 125.44, 128.19, 128.90, 129.33, 130.15, 130.76, 130.87, 132.69, 135.39, 142.69, 155.55, 155.83, 160.45, and 161.58. IR (KBr) cm^−^1: 1728. ESI-HRMS (*m/z*): calcd for C_25_H_19_FN_3_O_3_^+^ [M + H]^+^: 428.1405; found: 428.1402.

#### 7-((1-(4-Methylbenzyl)-1*H*-1,2,3-triazol-4-yl)methoxy)-4-phenyl-2*H*-chromen-2-one (7e)

mp 114–116 °C; yield 76%. ^1^H-NMR (DMSO-d_6_, 300 MHz): *δ* 2.31 (s, 3H, –CH_3_), 5.24 (s, 2H, –OCH_2_–), 5.53 (s, 2H, –CH_2_–), 6.15 (s, 1H, C=CH), 6.89 (d, 1H, *J* = 8.4 Hz, C=CH), 7.09 (s, 1H, C=CH), 7.13 (d, 1H, C=CH), 7.16–7.52 (m, 9H, Ar-H), 8.10 (s, 1H, Triazole-H). ^13^C-NMR (DMSO-d_6_, 75 MHz): *δ* 49.06, 62.06, 102.50, 111.97, 112.46, 113.46, 115.98, 116.25, 123.14, 123.33, 125.34, 125.71, 128.29, 128.91, 129.33, 130.15, 131.22, 135.39, 142.58, 155.56, 155.84, 160.45, and 161.58. IR (KBr) cm^−^1: 1724. ESI-HRMS (*m/z*): calcd for C_26_H_21_N_3_O_3_^+^ [M + H]^+^: 423.1577; found: 423.1568.

## Biological evaluation

### *In vitro* anti-proliferative activity

Human gastric cancer (AGS), human differentiation of advanced gastric cancer (MGC-803), human colorectal cancer (HCT-116), human lung cancer (A549), human liver cancer (HepG2), and human cervical cancer (HeLa) cell lines were obtained from the State Key Laboratory of Natural Resources and Functional Molecules of the Changbai Mountain (Yanbian University, Yanji, China) and maintained in Dulbecco’s modified Eagle’s medium (DMEM) and RPMI Media 1640 (RPMI1640), supplemented with 10% foetal bovine serum (FBS) 100 IU/ml penicillin, 100 mg/ml streptomycin, and 2 mmol/L L-glutamine (Sigma) at 37 °C in a humidified atmosphere containing 5% CO_2_.

Cells were plated in 96-well plates at appropriate densities to ensure exponential growth throughout the experimental period (9 × 10^3^ cells per well), and then allowed to adhere for 24 h. Cells were then treated for 48 h with four serial concentrations (1, 10, 50, and 100 µM) of each compound. 5-fluorouracil was used as a positive control. After 48 h of incubation, 10 µl of MTT solution were added to each well to a final concentration of 2 mg ml^−1^. Plates were then incubated for a further 4 h. After incubation, the MTT solution was removed and 150 µl of DMSO were added to each well for coloration. The plates were shaken vigorously for 10 min at room temperature to ensure complete solubilisation. The absorption was read on a microplate reader (ELx800, BioTek, Highland Park, Winooski, VT) at 492 nm, and the data were subsequently analysed. The results, which are expressed as the concentration of compound required to inhibit the cell growth by 50% (IC_50_), are summarised in Table 1.

### Analysis of the cell cycle distribution and apoptosis by flow cytometry

AGS cells were plated in 6-well plates (5.0 × 10^5^ cells per well) and incubated at 37 °C for 12 h. Exponentially growing cells were then incubated with compound **4d** at different concentrations (0, 10, and 100 µM). After 12 h, untreated cells (control) or cells treated with compound **4d** ware centrifuged at 1000 rpm (177 *g*) for 10 min, and then fixed in 70% ethanol at −20 °C for at least 24 h. The cells were subsequently resuspended in phosphate-buffered saline (PBS) containing 0.1 mg ml^−1^ RNase A and 5 µg ml^−1^ propidium iodide (PI). The cellular DNA content for the cell cycle distribution analysis was measured by flow cytometry using a FACSCalibur flow cytometer with Cell Quest software (Becton-Dickinson, Franklin Lakes, NJ), plotting at least 30,000 events per sample. The percentage of cells in the G0/G1, S, and G2/M phases of the cell cycle were determined using the ModFit LT version 4.0 software package (Verity Software, Topsham, ME).

Apoptosis was detected using an Apoptosis Detection Kit (Invitrogen, Eugene, OR). Briefly, cells were plated in 6-well plates (5.0 × 10^5^ cells per well) and incubated at 37 °C for 12 h. Exponentially, growing cells were then incubated with compound **4d** at different concentrations (0, 10, and 50 µM). Following 12 h of incubation, the cells were collected and washed twice with PBS, once with 1 × binding buffer, and then stained with 5 µM annexin V-FITC and 2.5 µM PI (5 mg ml^−1^) in 1 × binding buffer for 30 min at room temperature in the dark. Apoptotic cells were quantified using a FACSCalibur flow cytometer with the Cell Quest software (Becton-Dickinson, Franklin Lakes, NJ).

## Results and discussion

### Chemistry

**Scheme 1.**
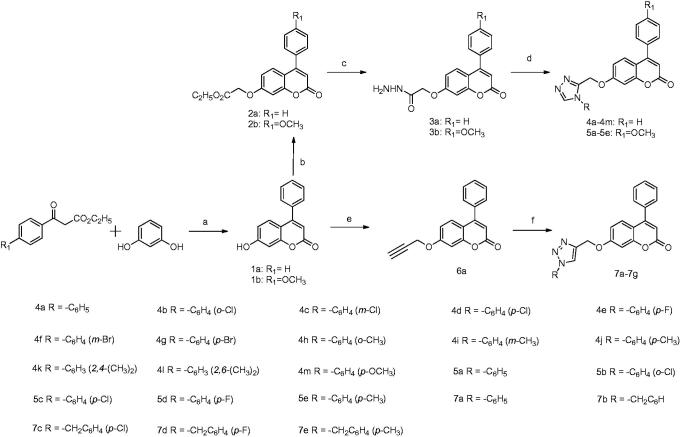
Reagents and conditions: (a) perchloric acid, rt; (b) K_2_CO_3_, ethyl bromoacetate, acetone, 56 °C; (c) hydrazine hydrate, ethyl alcohol, 78 °C; (d) (i) dimethylacetal, acetonitrile, 60 °C, 1 h; (ii) amines, glacial acetic acid, 120 °C; (e) K_2_CO_3_, propargyl bromide, acetone, 56 °C; (f) aromatic azides, CuSO_4_^.^5H_2_O, sodium ascorbate, t-BuOH/H_2_O (1:1), rt.

The synthetic procedure adopted to obtain the target compounds is shown in Scheme 1. The coumarin derivative **1a,b** was obtained by treatment of the condensation of resorcinol with beta-keto esters in perchloric acid, according to a slightly modified Pechmann’s method^21^. The new derivatives of compound **1a,b** were synthesised starting from a reaction with ethyl bromoacetate to give the corresponding esters, **2a,b**^22^. Hydrazinolysis of the latter afforded the acid hydrazides **3a,b**;^23^ which were condensed with N,N-dimethylformamide dimethyl acetal and aniline to furnish the target compounds, 3,4-disubstituted-1,2,4-triazoles, **4a–4m**, and **5a–5e**. Similarly, **1a** was subjected to alkylation at the hydroxyl position using propargyl bromide in the presence of K_2_CO_3_ (base) in acetone to form compound **6a**^24^. Derivative **6a** was allowed to undergo a 1,3-dipolar cycloaddition reaction typically called Huisgen cycloaddition with various aromatic azides under sharpless click chemistry conditions (CuSO_4_·5H_2_O and sodium ascorbate in t-BuOH/H_2_O (1:1)) to afford regioselectively 1,4-disubstituted-1,2,3-triazoles (**7a–7e**) in good to excellent yields. All of the synthesised compounds were assessed for their *in vitro* anti proliferative activity against all six human cancer cell lines.

## Biological evaluation

### *In vitro* cytotoxicity against human cancer cell lines

The *in vitro* antiproliferative activity of the newly synthesised derivatives was evaluated against all six human cancer cell lines using the MTT assay^25^. The clinically used antineoplastic drug 5-fluorouracil was used as the reference drug. The cytotoxicity profile determined in a previous study indicated that the parent molecule, 7-hydroxy-4-phenylchromen-2-one (**1a**), has no effect on cancer cell proliferation^26^. The present *in vitro* cytotoxicity study revealed that the synthesised compounds showed moderate to significant activity against all six cell lines. Interestingly, among the compounds tested, compound **4d** displayed the best activity against AGS. Of the 7-hydroxy-4-phenyl-2*H*-chromen-2-one derivatives of the 1,2,4-triazoles (**4a–4m**), compound **4d** exhibited potent activity against AGS, MGC-803, and HCT-116 cells, with IC_50_ values of 2.63 ± 0.17, 3.05 ± 0.29, and 11.57 ± 0.53 µM, respectively, and strong activity against the HeLa cell line, with an IC_50_ value of 13.62 ± 0.86 µM. Compound **4c** also exhibited strong antiproliferative activity against HCT-116 cells, with an IC_50_ value of 3.66 ± 0.24 µM. Almost all of the compounds strongly inhibited tumour proliferation. However, among the 7-hydroxy-4–(4-methoxyphenyl)-2*H*-chromen-2-one derivatives of the 1,2,4-triazoles (**5a–5e**), the coumarin derivatives exhibited greatly reduced activity against cancer cell lines, with compound **5a** exhibiting only moderate activity against the AGS cell line, with an IC_50_ value of 16.80 ± 0.83 µM. Interestingly, among the tested 1,2,4-triazole derivatives, compound **4 m** showed selective cytotoxic effects against the AGS cancer cell line, with an IC_50_ value of 9.00 ± 0.64 µM. Among the 1,2,3-triazoles (**7a–7e**), compound **7c** was the most active, with an IC_50_ of 48.16 ± 0.46 µM and selective activity against the MGC-803 cancer cell line. A family of 1,2,3-triazol derivatives was synthesised, but almost all of the compounds showed minimal effects on the proliferation of cancer cells.

### Structure activity relationship SAR

In view of the activity profile of the various compounds (Table 1), a structure activity relationship (SAR) was developed, which showed that the compounds with a methoxy group substitution at the R1 group in 1,2,4-triazole derivatives (**5a–5e**) were associated with a lack of cytotoxicity against the cancer cell lines examined. It was also observed that chlorine (Cl) and methyl (CH_3_) functionalities at the para-position play a significant role in enhancing the activity of the compound. It is necessary to point out that the cytotoxic activities of the derivatives (**4a–4 m**) with different halogen substitutions on the benzene ring were in the following order, p-Cl > o-Cl > m-Cl, p-CH_3_ > o-CH_3_ > m-CH_3_ and 2.4-(CH_3_)_2_ > 2.6-(CH_3_)_2_. Based on an overall comparison, the compounds derived from structures with electron-withdrawing substituents on the 1,2,4-triazole ring exhibited potent activity, and those with electron-donating substituents on the 1,2,4-triazole ring exhibited moderate activity, against the six cancer cell lines. However, compared with the activity of the 1,2,4-triazole derivatives, a complete loss of antiproliferative activity was observed when the 1,2,4-triazoles were replaced with the 1,2,3-triazoles (**7a–7e**) (Table 1).

### Cell cycle and annexin V/PI flow cytometry assays of derivative 4d

**Figure 2.**
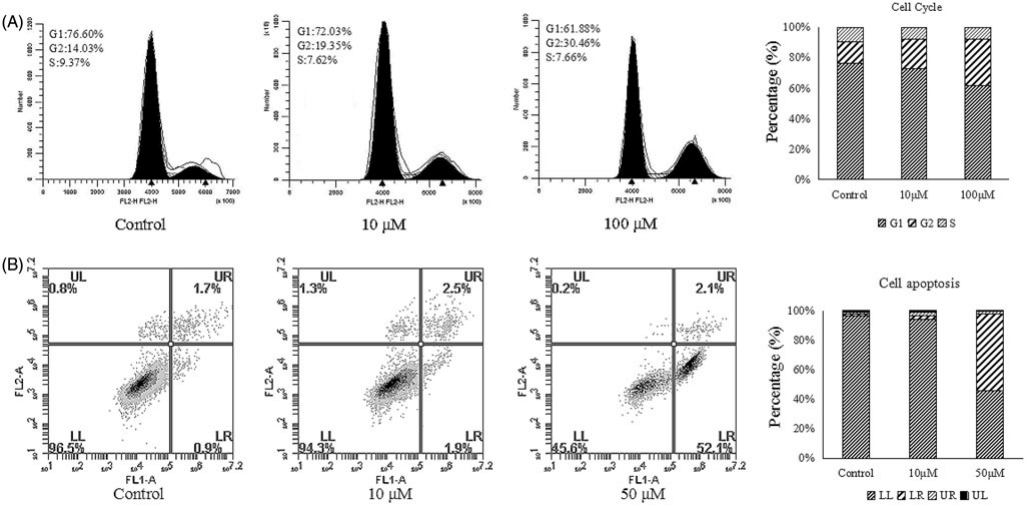
(A) Flow cytometry analyses of cell cycle distribution of gastric cancer cell AGS after treatment of compound **4d** (10 and 5 μM) and no treatment (Ctrl) as reference control for 12 h. (B) Apoptosis induction in lung carcinoma cell AGS after 12 h treatment with **4d** (10 and 50 μM) and no treatment.

Because compound **4d** showed the highest antiproliferative activity among all of the synthesised compounds, its effects on AGS cells were further assessed by flow cytometry assay using PI staining^27^. As seen in Figure 2, in comparison with the control group, treatment of the cells with compound **4d** (100 µM) led to a decrease in the percentage of cells in the G1 phase (from 76.60 to 61.88%) and a dramatic increase in cells in the G2 phase (from 14.03 to 30.46%), indicating that compound **4d** arrests cells in the G2/M phase of the cell cycle. To explore whether compound **4d** has the ability to induce apoptosis in AGS cells, we used annexin V-FITC and PI to stain cells and examined the staining by a flow cytometry analysis. The combined results of three independent experiments are depicted in Figure 2(B). AGS cells treated with 10 µM **4d** for 12 h showed an increase in the percentage of Annexin-V-positive cells, from 2.6% in control cells to 4.4% in treated cells (1.9% of cells in early apoptotic cells and 2.5% in late apoptotic cells). After increasing the concentration of the drug to 50 µM, the percentage of Annexin-V-positive cells increased to 54.2%, respectively. Our results suggest that compound **4d** induces apoptosis in AGS cells in a concentration-dependent manner.

## Conclusion

In this study, we have demonstrated the cytotoxic activity of a novel library of triazolyl derivatives of 7-hydroxy-4-phenylchromen-2-one anchored through the OH functionality at the C-7 position created through a regioselective click chemistry approach and characterised by a spectral data analysis. The new derivatives showed improved antiproliferative activities against several cancer cell lines compared with **1a**. Compound **4d** exhibited potent activity against AGS, MGC-803 and, HCT-116 cell lines, with IC_50_ values of 2.63 ± 0.17, 3.05 ± 0.29, and 11.57 ± 0.53 µM, respectively, and also had strong activity against the HeLa cell line, with an IC_50_ value of 13.62 ± 0.86 µM. The more detailed mechanistic study demonstrated that compound **4d** could inhibit the proliferation of AGS cancer cells by inducing apoptosis and arresting cells in the G2/M phase. Moreover, a structure activity relationship study revealed that the biological activity of the 1,2,4-triazole derivatives is significantly higher than that of the 1, 2, 3-triazole derivatives. Most of the compounds exhibited better antitumour activity than the positive control drug 5-fluorouracil.
